# Murine astrocytes produce IL-24 and are susceptible to the immunosuppressive effects of this cytokine

**DOI:** 10.1186/s12974-019-1444-1

**Published:** 2019-03-02

**Authors:** Amanda R. Burmeister, M. Brittany Johnson, Jessica J. Yaemmongkol, Ian Marriott

**Affiliations:** Department of Biological Sciences, University of North Carolina at Charlotte, 9201 University City Blvd., Charlotte, NC 28223 USA

**Keywords:** Interleukin-24, Interleukin-10, Astrocytes, Neuroinflammation, Bacterial infection

## Abstract

**Background:**

Glia are key regulators of inflammatory responses within the central nervous system (CNS) following infection or trauma. We have previously demonstrated the ability of activated glia to rapidly produce pro-inflammatory mediators followed by a transition to an anti-inflammatory cytokine production profile that includes the immunosuppressive cytokine interleukin (IL)-10 and the closely related cytokine IL-19. IL-24, another member of the IL-10 family, has been studied in a number of inflammatory conditions in the periphery and is known to modulate immune cell activity. However, the ability of glia to produce IL-24 remains unclear and the effects of this pleiotropic cytokine on glial immune functions have not been investigated.

**Methods:**

In this study, we have assessed whether primary murine glia produce IL-24 following stimulation and evaluated the effect of this cytokine on the immune responses of such cells. We have utilized RT-PCR and immunoblot analyses to assess the expression of IL-24 and its cognate receptors by astrocytes following challenge with bacteria or their components. Furthermore, we have determined the effect of recombinant IL-24 on astrocyte immune signaling and responses to clinically relevant bacteria using RT-PCR and specific capture ELISAs.

**Results:**

We demonstrate that astrocytes express IL-24 mRNA and release detectable amounts of this cytokine protein in a delayed manner following bacterial challenge. In addition, we have determined that glia constitutively express the cognate receptors for IL-24 and show that such expression can be increased in astrocytes following activation. Importantly, our results indicate that IL-24 exerts an immunosuppressive effect on astrocytes by elevating suppressor of cytokine signaling 3 expression and limiting IL-6 production following challenge. Furthermore, we have demonstrated that IL-24 can also augment the release of IL-10 by bacterially challenged astrocytes and can induce the expression of the potentially neuroprotective mediators, glutamate transporter 1, and cyclooxygenase 2.

**Conclusions:**

The expression of IL-24 and its cognate receptors by astrocytes following bacterial challenge, and the ability of this cytokine to limit inflammatory responses while promoting the expression of immunosuppressive and/or neuroprotective mediators, raises the intriguing possibility that IL-24 functions to regulate or resolve CNS inflammation following bacterial infection in order to limit neuronal damage.

## Background

Glial cells rapidly respond to invading pathogens by the production of an array of inflammatory mediators that include chemokines and cytokines. Such responses influence the integrity of the blood–brain barrier and serve to recruit leukocytes and activate them upon arrival at the site of infection [1, 2]. However, such responses can be detrimental if they are too severe or sustained, and this is of particular concern within the confines of the central nervous system (CNS). To limit the detrimental effects of inflammation, cell responses transition to a resolving phenotype that is typically characterized by a change in the cytokine production profile [3–6]. At peripheral sites, such compensatory anti-inflammatory responses are associated with the production of key immunosuppressive cytokines including IL-10 [7], and elevations in the level of this cytokine accompany host protection following bacterial or viral infection of the CNS [8, 9]. Indeed, we have previously demonstrated that both microglia and astrocytes show delayed expression of IL-10 following challenge with clinically relevant bacterial pathogens that functions to limit the inflammatory responses of these cells [10].

IL-10 acts on cells that express its heterodimeric receptor and elevates the expression of known anti-inflammatory response gene products, such as suppressor of cytokine signaling 3 (SOCS3). This molecule then inhibits the signaling cascade of members of the pro-inflammatory IL-6 cytokine family [11, 12]. However, it is now recognized that IL-10 is just one member of a family of cytokines that includes IL-19, IL-20, IL-22, IL-24, and IL-26 [13], which are grouped together based upon their structural homology and sharing of common receptor subunits. Unlike IL-10, the functions of these other family members are not as well defined in general, and their role in the CNS is largely unknown. Interestingly, we have demonstrated that IL-19, like IL-10, is expressed in a delayed manner by astrocytes following bacterial challenge and acts on glial cells in an immunosuppressive manner [14].

IL-24 (also known as melanoma differentiation associated gene 7; MDA-7) has been shown to be expressed in the CNS following RNA virus infection [15], but its function has not been determined. At peripheral sites, IL-24 has been shown to be pleiotropic with diverse functions depending on the target cell type and disease state [13]. It was first shown to induce melanoma cell apoptosis but has since been identified to contribute to both pro- and anti-inflammatory immune responses [16–19]. Like other members of the IL-10 family, IL-24 has been shown to be upregulated in patients suffering from disorders associated with chronic inflammation, including inflammatory bowel disease and psoriasis [13, 20, 21]. Furthermore, transgenic mice overexpressing IL-24 have been shown to develop psoriasis-like skin lesions, exhibiting thickening of the epidermis and monocyte infiltration [22, 23]. In contrast, *Staphylococcus aureus* skin infections in mice are associated with increased local IL-24 expression, and this cytokine was implicated in decreased levels of the pro-inflammatory cytokines IL-1β and IL-17 at sites of infection [21]. Furthermore, in the same study, it was demonstrated that IL-24 increases infection severity, consistent with an immunosuppressive role for this IL-10 family member [21].

In the present study, we have investigated the ability of primary murine glial cells to produce IL-24 and to respond to this cytokine. We demonstrate that astrocytes express IL-24 in a delayed manner in response to challenge with bacteria or their components. In addition, we have shown that glia constitutively express IL-24 receptors, and such expression is elevated in astrocytes following bacterial infection. Importantly, we have demonstrated that IL-24 inhibits the production of inflammatory cytokines by astrocytes and promotes the potentially neuroprotective functions of this cell type. Together, these data support a role for IL-24 in limiting detrimental inflammatory immune responses to CNS infection.

## Methods

### Bacterial propagation

*Neisseria meningitidis* strain MC58 (ATCC BAA-335) was grown on Columbia agar plates supplemented with 5% defibrinated sheep blood (BD, Franklin Lakes, NJ) and cultured in Columbia broth (BD Biosciences, San Jose, CA) on an orbital rocker at 37 °C with 5% CO_2_ overnight prior to in vitro challenge. A clinical isolate of *Streptococcus pneumoniae* strain CDC CS109 (ATCC 51915) was grown on commercially available trypticase soy agar with 5% sheep blood (BD Biosciences) and cultured overnight in tryptic soy broth in a similar manner to that described for *N. meningitidis*. *Staphylococcus aureus* strain UAMS-1 (ATCC 49230) was grown from frozen stock on lysogeny broth (LB) agar plates then cultured in tryptic soy broth overnight as described above. The number of colony forming units (CFU) for each bacterial species were determined by spectrophotometry using a Genespec3 spectrophotometer (MiraiBio Inc., Alameda CA).

### Intracranial bacterial administration

For in vivo experiments mice were uninfected or infected with *S. pneumoniae*. Bacteria was grown in a liquid culture prior to harvesting by centrifugation and washed in PBS. Three 6–8-week-old female C57BL/6J mice (Jackson Laboratories) were infected with bacteria via intracerebral (i.c.) injection of 1 × 10^7^ bacteria as previously described [14]. Mice were monitored and weighed twice per day and at 72 h post-infection, animals were euthanized, and whole brain tissue was isolated for analysis. Two female C57BL/6J mice were used as uninfected controls. All studies were performed in accordance with relevant federal guidelines and institutional policies regarding the use of animals for research purposes.

### Murine glial cell isolation and culture

Primary murine glial cells were isolated as described previously by our laboratory [1, 14, 24, 25]. Briefly, six to eight neonatal C57BL/6J mouse brains per preparation were dissected free of meninges and large blood vessels and finely minced with sterile surgical scissors. The minced tissue was then forced through a wire screen and briefly incubated with 0.25% trypsin 1 mM EDTA in serum-free RPMI 1640 medium for 5 min. The cell suspension was then washed, and this mixed glial culture was maintained in RPMI 1640 containing 10% fetal bovine serum (FBS) and penicillin-streptomycin mix for 2 weeks.

Astrocytes were isolated from mixed glial cultures by mild trypsinization (0.25% trypsin-1 mM EDTA for 20 min) in the absence of FBS as previously described [25, 26]. The remaining intact layer of adherent cells was demonstrated to be > 98% microglia by immunohistochemical staining for the microglial surface marker CD11b [25, 26], and the isolated astrocytes were determined to be > 96% pure based on morphological characteristics and the expression of the astrocyte marker glial fibrillary acidic protein (GFAP) as determined by immunofluorescence microscopy [26]. Microglia were maintained for 1 week in RPMI 1640 with 10% FBS and 20% conditioned medium from LADMAC cells (ATCC number CRL-2420), a murine monocyte-like cell line that secretes colony stimulating factor-1 (CSF-1) [14], while astrocytes were cultured in RPMI 1640 containing 10% FBS. All studies were performed in accordance with relevant federal guidelines and institutional policies regarding the use of animals for research purposes.

### In vitro bacterial infection of isolated glial cells and exposure to bacterial components and recombinant IL-24

Glial cells were exposed to bacteria at multiplicities of infection (MOI) of 1:1, 1:10, or 1:50 glia to bacteria in antibiotic-free medium for 2 h at 37 °C with 5% CO_2_. These doses were employed as these bacterial numbers are within the range previously reported for the cerebral spinal fluid of children with bacterial meningitis [27]. Following this incubation period, complete RPMI 1640 media supplemented with 10% FBS and penicillin-streptomycin (MilliporeSigma, St. Louis, MO) was added to kill extracellular bacteria [14]. Alternatively, glial cells were exposed to bacterial lipopolysaccharide (LPS) isolated from *Escherichia coli* (MilliporeSigma), Pam3Cys-Ser-(Lys)4 (Pam3Cys; InvivoGen, San Diego, CA), bacterial flagellin isolated from *Salmonella typhimurium* strain 14028 (Enzolife Sciences, Farmingdale, NY), or polyinosinic–polycytidylic acid (polyI:C; MilliporeSigma). In some studies, glial cells were also treated with commercially available recombinant murine IL-24 protein (R&D Systems, Minneapolis, MN) at concentrations of 10, 30, or 100 ng/ml. At the indicated time points following challenge and/or IL-24 treatment, whole cell protein lysates were collected and RNA was isolated for immunoblot analysis and RT-PCR, respectively.

### RNA extraction and semi-quantitative reverse transcription PCR (RT-PCR)

Total RNA was isolated from cultured glial cells using Trizol Reagent (Thermo Fisher Scientific) according to the manufacturer’s instructions and quantified using a Nanodrop ND-1000 spectrophotometer. All RNA samples were diluted to the same concentration and reverse transcribed in the presence of random hexamers using 200 U of RNase H minus Moloney leukemia virus reverse transcriptase (Promega, Madison, WI) in the buffer supplied by the manufacturer. Semi-quantitative RT-PCR was performed on 5% of the reverse-transcribed cDNA product to assess the relative levels of expression of mRNA-encoding IL-24, interleukin 22 receptor α (IL-22Rα), cyclooxygenase 2 (COX2), glutamate transporter 1 (GLT-1), suppressor of cytokine signaling 3 (SOCS3), and the housekeeping gene glyceraldehyde 3-phosphate dehydrogenase (GAPDH). Primers were designed spanning multiple exons using either Primer-BLAST (National Center for Biotechnology Information, Bethesda, MD) or Primer3 web interface [28] and are shown in Table 1. RT-PCR products were separated by electrophoresis on 1.5% agarose gels and imaged using Bio-Rad EZ imaging system and densitometric analysis was performed using ImageLab software (Bio-Rad, Hercules, CA). In addition, real-time RT-PCR was performed to quantify IL-24 mRNA expression using a QuantiTect SYBR Green approach (Qiagen, Valencia, CA) on a 7500 Fast Real-Time PCR machine (Life Technologies) according to the manufacturer’s protocol and as described previously by our laboratory [25].

**Table 1:** PCR primer sequences utilized

mRNA	Forward primer (5′-3′)	Reverse primer (5′-3′)
IL-24	CTGGACTGTGAAGAACACTGTGCA	GTCCAGCCCAAAGGCTTTCAC
IL-22Rα	TGACTGATCGTTTCAGCTCGCTGC	GGAGTCAGGCCAAGGAACTCGTAT
COX2	TCAGCCAGGCAGCAAATCCTTG	TAGTCTCTCCTATGAGTATGAGTC
GLT-1	CAAGTCTGAGCTGGACACCA	GGCTGAGAATCGGGTCATTA
SOCS3	TTTCGCTTCGGGACTAGC	CGCTCAACGTGAAGAAGTG
GAPDH	CCATCACCATCTTCCAGGAGCGAG	CACAGTCTTCTGGGTGGCAGTGAT

### Immunoblot analysis

Immunoblot analyses for the presence of secreted IL-24 in cell culture medium and IL-22Rα, phospho-signal transducer and activator of transcription 1 (pSTAT1), pSTAT3, and SOCS3 in whole cell protein isolates were performed as described previously by our laboratory [1, 10, 29]. After incubation with a rat monoclonal IgG antibody directed against mouse IL-24 (Clone 303308; R&D Systems), a rat monoclonal IgG for murine IL-22Rα (Clone 496504; R&D Systems), a rabbit monoclonal IgG antibody directed against mouse pSTAT1 (Clone 58D6; Cell Signaling), a rabbit monoclonal IgG antibody directed against mouse pSTAT3 (Clone D3A7; Cell Signaling), or a mouse monoclonal IgG directed against mouse SOCS3 (Clone 1B2; MilliporeSigma) for 24 h at 4 °C, blots were washed and incubated in the presence of appropriate horseradish peroxidase-conjugated secondary antibodies. Bound enzyme was detected with Advansta Western Bright enhanced chemiluminescence reagent (Advansta, Menlo Park, CA) with a Bio-Rad ChemiDoc imaging system (Bio-Rad, Hercules, CA). To assess total protein loading in each well, immunoblots were re-probed with a mouse monoclonal antibody directed against β-actin (Abcam, Cambridge MA) or an irrelevant protein on Coomassie Blue stained gels was used as a loading control. Immunoblots shown are representative of at least three separate experiments and ImageLab software (Bio-Rad) was used for densitometric analysis. IL-24, IL-22Rα, and SOCS3 levels are reported relative to levels in unstimulated cells normalized to β-actin expression.

### Quantification of IL-6, IL-10, and TNF-α in glial cell culture supernatants

Specific capture ELISAs were performed to quantify the release of murine IL-6, IL-10, and TNF-α. Commercially available Duoset® ELISA kits were used to measure IL-10 and TNF-α secretion (R&D Systems), while murine IL-6 secretion was measured using a rat anti-mouse IL-6 capture antibody (Clone MP5-20F3) and a biotinylated rat anti-mouse IL-6 detection antibody (Clone MP5-C2311) (BD Biosciences). Bound antibody was detected by addition of streptavidin-horseradish peroxidase (BD Biosciences). After addition of TMB substrate and H_2_SO_4_ stop solution, absorbances were measured at 450 nm using a Tecan Sunrise™ (Tecan Group, Männedorf, Switzerland) microplate reader. A standard curve was constructed using varying dilutions of recombinant cytokines (BD Biosciences), and the cytokine content of culture supernatants determined by extrapolation of absorbances to the standard curve.

### Cell viability assay

Cell viability was assessed at 48 h following treatment with recombinant IL-24 and/or bacterial challenge with a CellTiter96®AQ_ueous_ cell proliferation assay (3-(4,5-dimethylthiazol-2-yl)-5-(3-carboxymethoxyphenyl)-2-(4-sulfophenyl)-2H-terazolium, MTS) according to the manufacturer’s protocol (Promega, Madison, WI), and absorbance values were quantified using a microplate reader at 490 nm. As a positive control, glial cells were treated with 0.1% Triton X-100.

### Statistical analysis

Data is presented as the mean ± standard error of the mean (SEM). Statistical analyses were performed using Student’s *t* test or one-way analysis of variance (ANOVA) with Tukey’s post hoc test using commercially available software (GraphPad Prism, GraphPad Software, La Jolla, CA). In all experiments, results were considered statistically significant when a *P* value of less than 0.05 was obtained.

## Results

### IL-24 is expressed by murine glial cells following bacterial stimulation

**Fig. 1:**
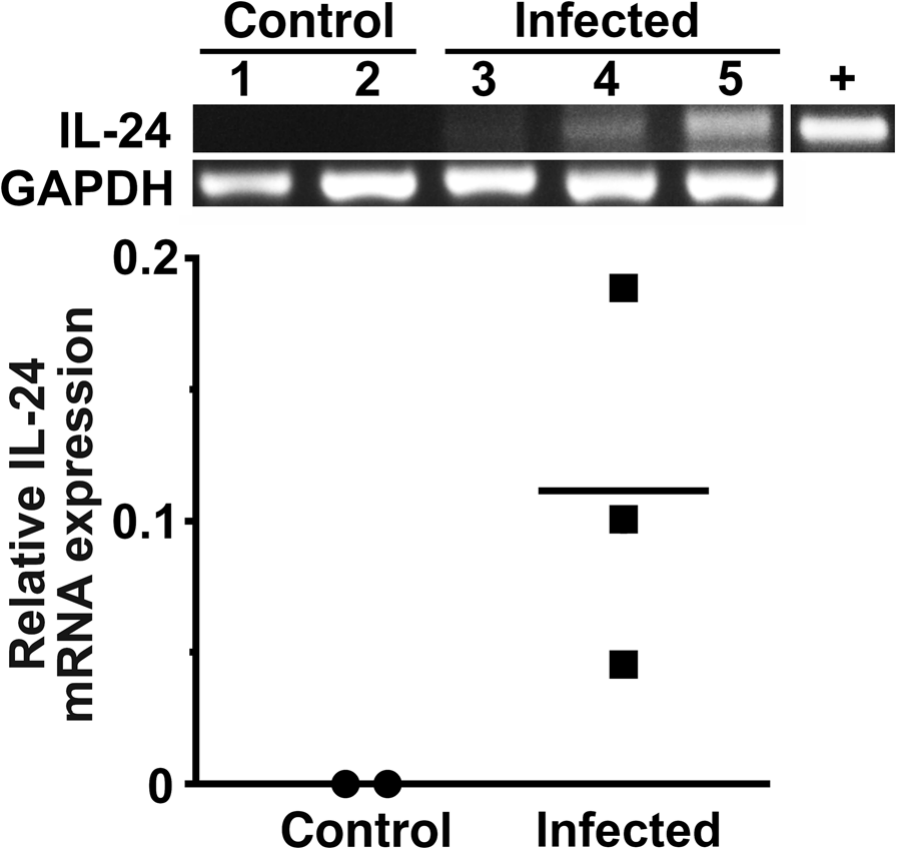
IL-24 mRNA expression is induced in the CNS of mice following in vivo bacterial infection. Wild type C57BL/6J mice were uninfected (control, animal numbers 1 and 2) or infected by direct intracranial administration with *Streptococcus pneumoniae* (1 × 10^7^ bacteria, animal numbers 3–6). At 72 h following infection, whole brain tissue was collected and expression of mRNA encoding for IL-24 was determined by semi-quantitative RT-PCR, and C57BL/6J mouse whole thymus tissue was used as a positive control (+). Relative IL-24 mRNA expression was determined by densitometric analysis and normalized to the level of the housekeeping gene GAPDH, and all data points and the mean are shown

To begin to assess whether cells within the CNS can express IL-24, we have determined whether mRNA encoding IL-24 is present in the CNS either constitutively or following bacterial infection. As shown in Fig. 1, mRNA encoding IL-24 was not detectable in the brains of uninfected mice as determined by semi-quantitative RT-PCR. However, IL-24 mRNA expression, albeit with high variability, was discernable in the brains of all infected animals at 72 h following direct intracranial bacterial administration (Fig. 1).

**Fig. 2:**
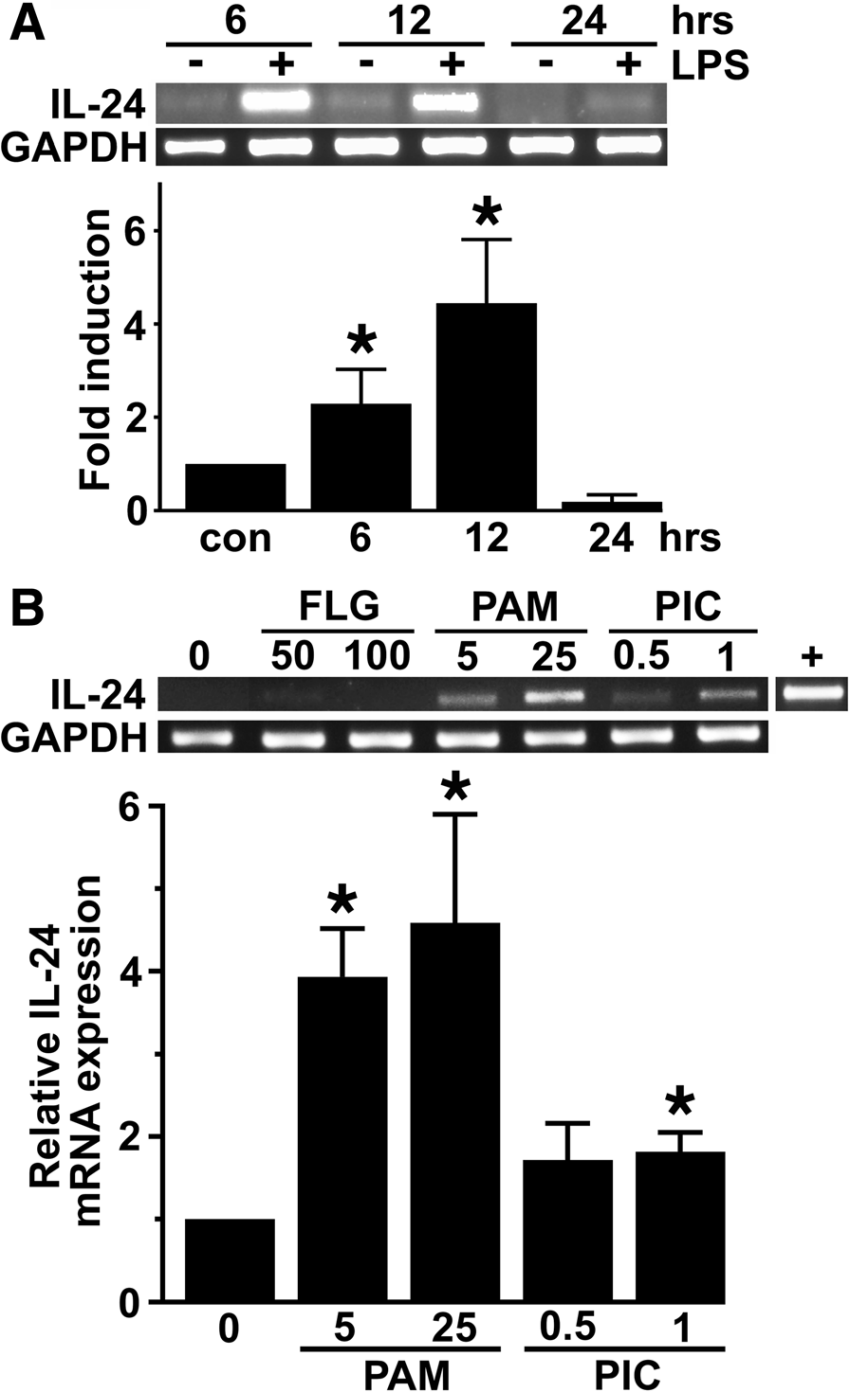
Primary murine astrocytes express IL-24 following exposure to bacterial ligands for certain toll-like receptors. **a** Murine astrocytes were either unstimulated or challenged with LPS (5 ng/ml) for 6, 12, or 24 h, and IL-24 mRNA expression was determined by semi-quantitative (top) and real-time quantitative (bottom) RT-PCR. Expression of GAPDH mRNA housekeeping gene product is included and the image shown is representative of at least three independent experiments. Below, real-time RT-PCR data is shown as mean fold increases in product ± the SEM of three independent experiments and an asterisk indicates a statistically significant difference from unchallenged cells (*p* < 0.05). **b** Astrocytes were either unchallenged or challenged with TLR ligands; flagellin (50 and 100 ng/mL; FLG), the lipoprotein Pam3Cys (5 and 25 ng/mL; PAM), or dsRNA polyinosinic:polycytidylic acid (0.5 or 1 μg/mL; PIC) for 6 h, and IL-24 mRNA expression was determined by semi-quantitative RT-PCR. Expression of the housekeeping gene GAPDH is shown, and relative IL-24 mRNA expression was determined by densitometric analysis and normalized to unchallenged cells. Murine whole thymus tissue was used as a positive control for IL-24 expression (+). Asterisks denote statistical significance compared to unchallenged cells (*p* < 0.05)

**Fig. 3:**
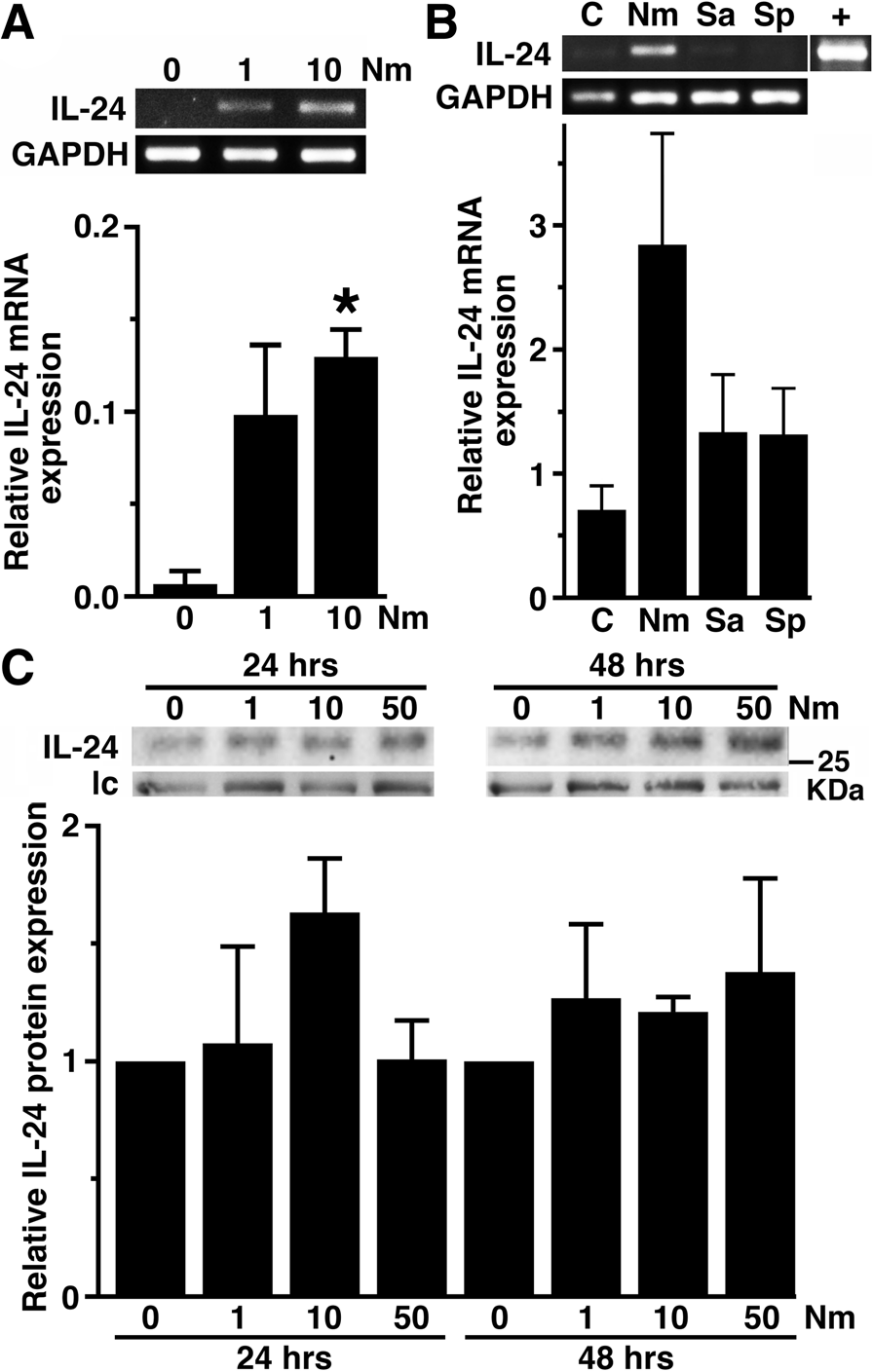
Primary murine glia express IL-24 following bacterial challenge. **a** Astrocytes were uninfected or infected with *Neisseria meningitidis* (Nm) at MOI of 1 or 10 bacteria to glia for 6 h prior to RNA collection. IL-24 and GAPDH mRNA expression was determined by semi-quantitative RT-PCR, and the average of three separate experiments is shown as mean relative gene expression as determined by densitometric analysis normalized to the expression of the housekeeping gene GAPDH ± SEM. **b** Isolated primary murine microglia were uninfected or infected with *N*. *meningitidis*, *Staphylococcus aureus* (Sa), or *S. pneumoniae* (Sp) (MOI of 10:1 bacteria to microglia) for 8 h prior to RNA isolation. C57BL/6J thymus tissue was used as a positive control. Relative IL-24 gene expression was determined by densitometric analysis normalized to GAPDH gene expression and is depicted as the mean of three individual experiments ± SEM. **c** Astrocytes were uninfected or infected with *N. meningitidis* for 24 or 48 h prior to immunoblot analysis of cell medium IL-24 protein content. Expression of an irrelevant protein is shown as a loading control (lc), and the relative IL-24 expression was determined by densitometric analysis and normalized to untreated cells. Data is expressed as the mean ± the SEM of three independent experiments. Asterisk indicates a statistical significance compared to unchallenged cells (*p* < 0.05)

To determine whether the presence of IL-24 mRNA in the brain following in vivo infection is due to the expression of this cytokine by glial cells, we have assessed the in vitro IL-24 expression in isolated primary murine astrocytes and microglia by semi-quantitative and quantitative real-time RT-PCR. As shown in Fig. 2a, murine astrocytes constitutively express low levels of IL-24 mRNA but challenge with bacterial LPS elicited marked increases in IL-24 mRNA expression. Such a response was not limited to this TLR4 ligand as 6-h exposure to the TLR2 ligand Pam3Cys and, to a lesser extent, the TLR3 ligand polyI:C also elicited significant increases in IL-24 mRNA levels in astrocytes (Fig. 2b). However, the TLR5 ligand, flagellin, failed to induce IL-24 mRNA expression suggesting at least some specificity in the IL-24 mRNA responses to TLR ligands (Fig. 2b). In addition, we have determined that exposure to intact viable bacteria can similarly induce IL-24 expression with the demonstration that *N. meningiditis* elicits a significant increase in IL-24 mRNA levels in astrocytes at 6 h following bacterial challenge (Fig. 3a). Similar to astrocytes, murine microglia constitutively express little to no mRNA encoding IL-24, but exposure to bacteria elicits discernable increases in IL-24 mRNA expression, although the responses to Gram-positive species *S. aureus* and *S. pneumoniae* were far more modest than that seen for the Gram-negative organism *N. meningitidis* (Fig. 3b).

Consistent with the low levels of IL-24 mRNA expression observed, resting astrocytes (Fig. 3c) and microglia (data not shown) demonstrated only limited IL-24 protein production as determined by immunoblot analysis. Exposure to *N. meningitidis* induced detectable, but highly variable, increases in IL-24 protein production by astrocytes that approached statistical significance at 24 h following bacterial challenge (Fig. 3c), with approximately 400 pg/ml produced as estimated by comparison with immunoblots of standards at known concentrations. Interestingly, no such increases in IL-24 protein production were detectable in murine microglia (data not shown).

### Primary murine glia express IL-24 receptor subunits

**Fig. 4:**
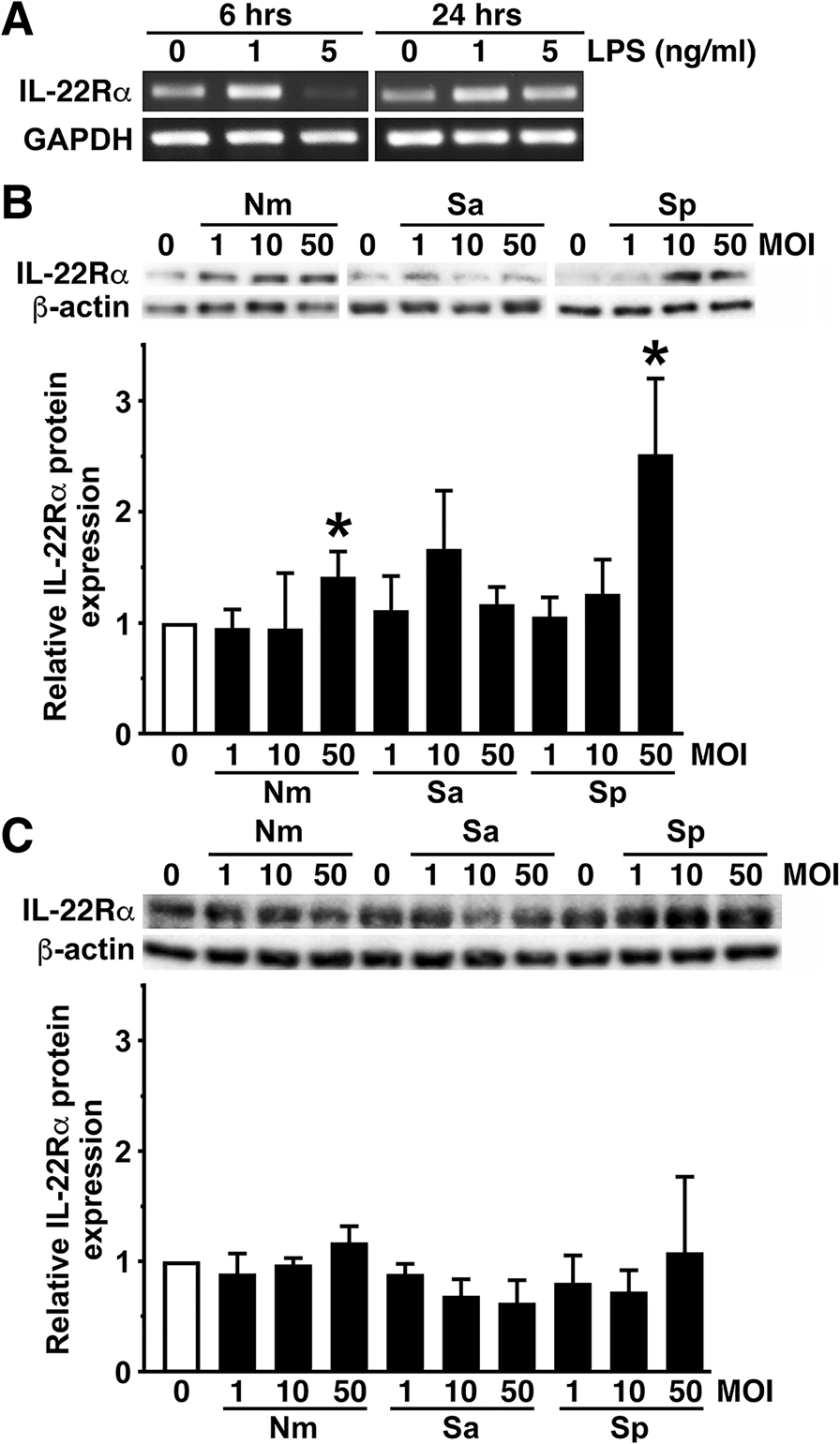
Primary murine glia constitutively express the IL-22Rα subunit of the Type II receptor for IL-24. **a** Murine astrocytes were unstimulated or challenged with LPS (1 or 5 ng/ml) for 6 or 24 h, and levels of mRNA encoding IL-22Rα and GAPDH were determined by semi-quantitative RT-PCR. **b** Astrocytes were uninfected or infected with *N. meningitidis* (Nm), *S. aureus* (Sa), or *S. pneumoniae* (Sp) at MOI of 1, 10, or 50 bacteria to glia for 24 h prior to immunoblot analysis for IL-22Rα expression. Expression of β-actin is shown as a loading control, and relative IL-22Rα expression was determined by densitometric analysis and normalized to untreated cells. Data is expressed as the mean ± the SEM of 3 independent experiments, and an asterisk indicates a statistically significant difference from unchallenged cells (*p* < 0.05). **c** Murine microglia were uninfected or infected with *N. meningitidis* (Nm), *S. aureus* (Sa), or *S. pneumoniae* (Sp) at MOI of 1, 10, or 50 bacteria to glia for 24 h prior to immunoblot analysis for IL-22Rα expression. Expression of β-actin is shown as a loading control and relative IL-22Rα expression was determined by densitometric analysis and normalized to untreated cells. Data is expressed as the mean ± the SEM of three independent experiments

IL-24 elicits cellular responses via Type I and Type II receptors that are composed of IL-20Rα/IL-20Rβ and IL-22Rα/IL-20Rβ subunits, respectively [30]. We have previously demonstrated that murine astrocytes can express both IL-20Rα and IL-20Rβ, while microglia express IL-20Rβ but not IL-20Rα [14]. Here, we have determined whether murine glia also express IL-22Rα. As shown in Fig. 4a, primary murine astrocytes constitutively express mRNA encoding IL-22Rα and have low level protein expression of this receptor subunit at rest. However, such expression was elevated following stimulation with LPS (Fig. 4a) or challenge with *N. meningiditis* or *S. pneumoniae* (Fig. 4b). Interestingly, and in contrast to IL-20Rα, primary murine microglia constitutively expressed robust levels of IL-22Rα protein, and such expression was not elevated further following challenge with either Gram-negative or Gram-positive bacterial species (Fig. 4c).

### IL-24 augments the expression of suppressive cytokine signaling components and limits inflammatory cytokine production by activated astrocytes

**Fig. 5:**
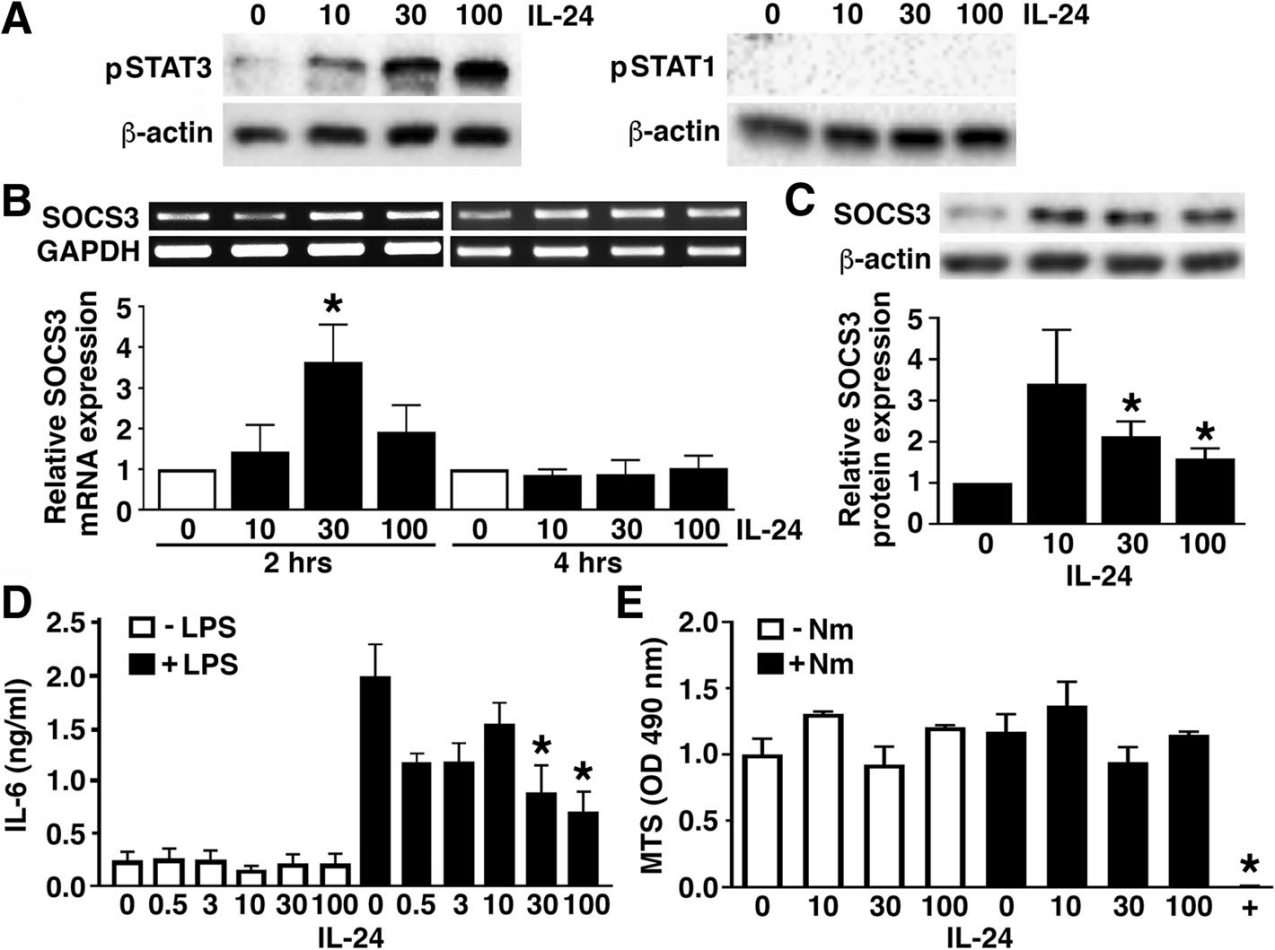
IL-24 augments the expression of suppressive cytokine signaling components by murine astrocytes and limits inflammatory cytokine release by these cells. **a** Astrocytes were untreated or treated with recombinant IL-24 (10, 30, or 100 ng/mL) for 30 min and the presence of phosphorylated STAT3 and STAT1 was determined by immunoblot analysis. Expression of β-actin is shown as a loading control and these immunoblots are representative of two separate experiments. **b** Astrocytes were untreated or treated with recombinant IL-24 (10, 30, or 100 ng/mL) for 2 or 4 h, and SOCS3 mRNA expression was determined by semi-quantitative RT-PCR. Expression of the housekeeping gene product GAPDH is shown, and relative SOCS3 expression was determined by densitometric analysis and normalized to untreated cells. Data is expressed as the mean ± the SEM of three independent experiments, and an asterisk indicates a statistically significant difference from unchallenged cells at each time point (*p* < 0.05). **c** Astrocytes were untreated or treated with recombinant IL-24 (10, 30, or 100 ng/mL) for 8 h prior to immunoblot analysis for SOCS3 protein expression. Expression of the housekeeping gene β-actin is shown and relative SOCS3 protein expression was determined by densitometric analysis normalized to untreated cells. Asterisks indicate statistically significant differences from unchallenged cells (p < 0.05). **d** Astrocytes were untreated or treated with IL-24 (0.5, 3, 10, 30, or 100 ng/mL) for 4 h prior to challenge with bacterial LPS (5 ng/mL) or vehicle control for 12 h, and IL-6 secretion was determined by specific capture ELISA. Asterisks indicate a statistically significant difference (*p* < 0.05) from similarly challenged cells in the absence of IL-24 (*n* = 3). **e** Primary astrocytes were untreated or treated with recombinant IL-24 (10, 30, or 100 ng/mL) for 4 h prior to being uninfected or infected with Nm for 48 h before cell viability analysis via MTS assay. Data is presented as the mean absorbance ± SEM for three experiments. 0.1% Triton X-100 was used as a positive control, and an asterisk indicates a statistically significant difference from unchallenged cells in the absence of IL-24 (*p* < 0.05)

Having established the ability of astrocytes to express receptors for IL-24, we next assessed the effects of this cytokine on astrocyte immune functions. As shown in Fig. 5a, treatment of astrocytes with recombinant IL-24 elicited the activation of STAT3, but not STAT1, within 30 min. Similarly, rIL-24 elicited a rapid but transient increase in the expression of mRNA encoding the immunosuppressive signaling component SOCS3 in these cells (Fig. 5b). Importantly, treatment with IL-24 for 8 h induced significant increases in SOCS3 protein expression in astrocytes (Fig. 5c). In addition, while 12- or 18-h IL-24 treatment failed to elicit production of IL-6 (Fig. 5d) or TNF-α (data not shown) by these glial cells, it significantly inhibited the production of this inflammatory mediator by astrocytes at 12 h following LPS challenge (Fig. 5d) and reduced LPS-induced TNF-α production, although this effect was not statistically significant at this time point (data not shown). This effect was not attributable to changes in astrocyte viability as treatment with up to 100 ng/ml IL-24 for 48 h failed to elicit significant effects on cell viability as assessed by MTS assay (Fig. 5e). Together, these data are consistent with an ability of IL-24 to suppress astrocyte inflammatory responses.

### IL-24 increases anti-inflammatory/neuroprotective mediator expression by astrocytes

**Fig. 6:**
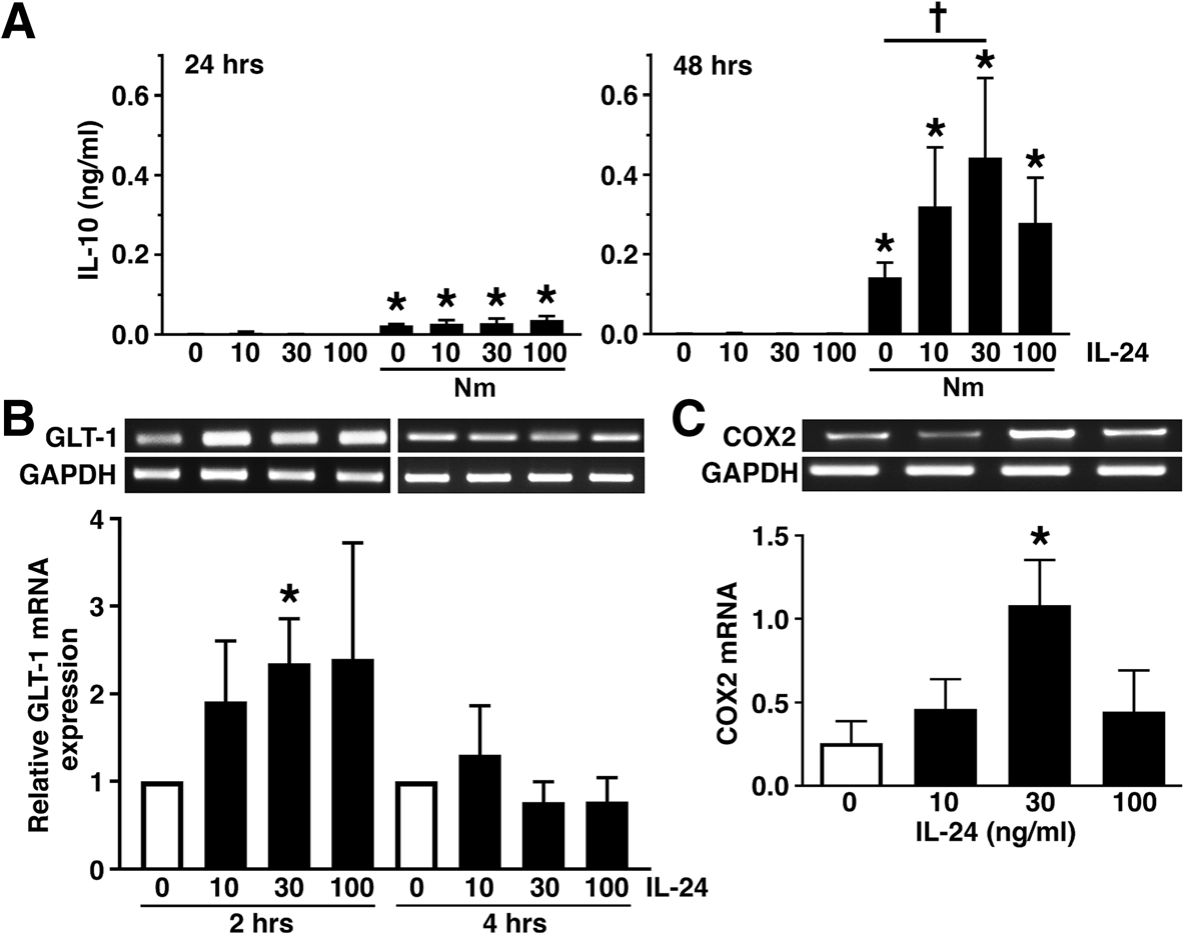
IL-24 increases the expression of anti-inflammatory cytokines and neuroprotective factors by primary murine astrocytes. **a** Astrocytes were untreated or treated with recombinant IL-24 (10, 30, or 100 ng/ml) for 4 h prior to *N. meningitidis* infection (Nm; MOI of 10:1 bacteria to each astrocyte) or vehicle control. At 24 or 48 h postinfection, IL-10 protein release was assessed by specific capture ELISA. Asterisks and dagger indicate a statistically significant difference from uninfected cells and similarly challenged cells in the absence of IL-24, respectively (*n* = 3; *p* < 0.05). **b** Astrocytes were untreated or treated with recombinant IL-24 (10, 30, or 100 ng/mL) for 2 or 4 h, and mRNA expression of GLT-1 was determined by semi-quantitative RT-PCR. Expression of the housekeeping gene product GAPDH is shown, and relative GLT-1 expression was determined by densitometric analysis and normalized to untreated cells. Data is expressed as the mean ± the SEM of three independent experiments, and an asterisk indicates a statistically significant difference (*p* < 0.05) from unchallenged cells at each time point. **c** Astrocytes were untreated or treated with recombinant IL-24 (10, 30, or 100 ng/mL) for 2 h and mRNA expression of COX2 was determined by semi-quantitative RT-PCR. Data is expressed as the mean ± the SEM of three independent experiments, and an asterisk indicates a statistically significant difference (*p* < 0.05) from unchallenged cells

To further determine whether IL-24 promotes inflammatory or protective murine astrocyte responses, we assessed the effects of this cytokine on the expression of the immunosuppressive factor IL-10. As shown in Fig. 6a, *N. meningitidis* elicits the delayed production of IL-10, with low but detectable levels of this cytokine at 24 h following infection consistent with our previous studies [10]. In contrast, IL-24 treatment alone failed to elicit IL-10 production by astrocytes at either 24 or 48 h (Fig. 6a). Importantly, this cytokine significantly elevated the level of IL-10 release by this cell type at 48 h after challenge with *N. meningitidis* (Fig. 6a). We confirmed that such an effect was not due to differences in cell number or survival following bacterial challenge by MTS assay (Fig. 5d). Interestingly, we determined that IL-24 treatment also elevates the expression of mRNA encoding GLT-1 (Fig. 6b), a transporter for the potentially cytotoxic neurotransmitter glutamate, and COX2 (Fig. 6c), the enzyme responsible for the production of prostaglandins that can act in an anti-inflammatory manner. Together, these data are consistent with an ability of IL-24 to promote protective/anti-inflammatory astrocyte responses to bacterial pathogens.

## Discussion

There is growing appreciation that activated glial cells not only initiate inflammatory responses within the CNS, but also play a critical role in limiting sustained and/or excessive neuroinflammation. During the resolution phase of normal host immune responses at peripheral sites, immunosuppressive cytokines and tissue repair factors are produced that serve to prevent inflammatory damage [7]. Consistent with this, we have demonstrated that glial cells produce the immunosuppressive cytokines IL-10 and IL-19 in a delayed manner following bacterial challenge [10, 14]. In the present study, we demonstrate that primary murine astrocytes can express another member of the IL-10 family of cytokines, IL-24, at the level of both mRNA expression and protein release, in a delayed manner following exposure to bacteria or certain bacterial components. Such a finding is consistent with one published study by Das and coworkers [15] that showed expression of mRNA encoding this cytokine by murine astrocytes following infection with Chikungunya virus.

In a previous study, we showed that murine and human astrocytes constitutively express both the IL-20Rα and IL-20Rβ heterodimeric subunits of the Type I IL-24 receptor [14]. Here, we have demonstrated that murine astrocytes also constitutively express the IL-22Rα subunit that, together with IL-20Rβ, constitutes the Type II receptor for this cytokine [13, 30–32]. Interestingly, such constitutive expression can be elevated at the level of mRNA or protein expression by exposure to LPS or bacterial infection. These findings are consistent with an earlier study demonstrating that immortalized human fetal astrocytes and a glioblastoma cell line express mRNA encoding the three subunits that comprise the Type I and II IL-24 receptors [33]. Such expression is in contrast with microglia that we have shown to express the IL-20Rβ subunit but lack IL-20Rα and so fail to express the Type I IL-24 receptor [14]. However, in the present study, we have demonstrated that microglia constitutively express robust levels of the IL-22Rα subunit protein that cannot be elevated further following bacterial challenge. As such, this glial cell type can express the Type II IL-24 receptor, and studies to assess the effect of this cytokine on microglial functions are ongoing.

IL-24, like IL-10, has been reported to exert pleiotropic effects that include an ability to both promote and inhibit inflammation at peripheral sites such as the skin [13, 21, 32]. In our previous studies, we have demonstrated that IL-10 and IL-19 can limit inflammatory mediator production by astrocytes following exposure to clinically relevant bacterial pathogens [10, 14]. While the delayed production of IL-24 by activated astrocytes is similar to the production of IL-10 and IL-19 by these cells following infection [10, 14] and is consistent with a role in infection resolution, we have directly assessed the effect of this cytokine on inflammatory astrocyte responses.

We have previously demonstrated that the immunosuppressive effects of IL-10 and IL-19 in astrocytes are associated with the activation of STAT3 and the induction of expression of the feedback inhibitor, SOCS3, which is a key inhibitor in the pro-inflammatory IL-6 signaling cascade [10, 14]. SOCS3 acts by directly inhibiting the Janus kinase/signal transducer and activator of transcription (JAK/STAT) pathway of IL-6 by negatively regulating gp130-mediatiated STAT3 activation [12]. Here, we have shown that IL-24 elicits rapid STAT3 activation in astrocytes and also induces the expression of SOCS3 in astrocytes, suggesting that IL-24 could similarly play a role in limiting the inflammatory signaling of IL-6 in this cell type. Furthermore, we have shown that IL-24 fails to induce either IL-6 or TNF-α production by unstimulated astrocytes but can significantly reduce IL-6 release by LPS challenged cells. Such a finding is similar to the actions of IL-10 and IL-19 on activated astrocytes [10, 14] and is consistent with an immunosuppressive effect of IL-24 on this resident CNS cell type.

To further determine whether IL-24 exerts pro-inflammatory or immunosuppressive effects on astrocytes, we have investigated the ability of this cytokine to promote responses that could limit inflammatory damage and/or protect neuronal function. We have found that, while IL-24 does not promote the release of the immunosuppressive cytokine IL-10 by unstimulated astrocytes, it can significantly augment the delayed production of this cytokine by cells following bacterial challenge. Interestingly, we have shown that IL-24 can also upregulate the expression of other molecules that could be neuroprotective. GLT-1 functions to reduce the level of free extracellular glutamate, and protects neurons from excitotoxicity associated with excessive or sustained elevations in the extracellular levels of this neurotransmitter [34–36]. Here, we have shown that IL-24 can elicit a rapid increase in the level of expression of this transporter offering another potential mechanism by which this cytokine could confer neuroprotection. Finally, we have determined that IL-24 can also elicit a rapid elevation in the expression of COX2, an enzyme that is critically important for the production of a variety of prostaglandins. While an induction in COX2 expression and activity is often associated with inflammation, the roles of these mediators are notoriously difficult to attribute definitively. Indeed, numerous studies have described the ability of prostaglandins such as PGD2 and even PGE2, to suppress the inflammatory responses of glial cells [37–42]. As such, it is possible that the ability of IL-24 to upregulate COX2 expression could result in the production of prostaglandins that function to suppress inflammation, although further studies will be required to confirm this hypothesis.

## Conclusions

Taken together, these studies have determined that primary astrocytes can express IL-24 in a delayed manner in response to bacterial challenge. Furthermore, this major glial cell population is responsive to this novel IL-10 family member as it expresses the subunits that constitute both cognate Type I and Type II receptors for IL-24. Importantly, our results indicate that IL-24, like IL-10 and IL-19, may function to limit the inflammatory responses of astrocytes to bacterial pathogens while promoting the expression of anti-inflammatory and potentially neuroprotective mediators by this resident CNS cell type. As such, the present study supports the notion that IL-24 production by astrocytes and/or infiltrating leukocytes could function to regulate or resolve CNS inflammation following infection in order to limit neuronal damage. However, it remains to be definitively established whether the effects of IL-24 are direct or indirect, or occur alone or in combination with other cytokines, and further studies are clearly warranted to assess the effects of IL-24 on host responses in vivo to clinically relevant bacterial pathogens of the CNS.

## Abbreviations

ANOVA: Analysis of variance
CFU: Colony forming units
CNS: Central nervous system
COX2: Cyclooxygenase 2
ELISA: Enzyme linked immunosorbent assay
GAPDH: Glyceraldehyde 3-phosphate dehydrogenase
GFAP: Glial fibrillary acidic protein
GLT-1: Glutamate transporter-1
IACUC: Institutional Animal Care and Use Committee
IL: Interleukin
LB: Lysogeny broth
MDA-7: Melanoma differentiation-associated gene 7
MOI: Multiplicity of infection
mRNA: Messenger ribonucleic acid
MTS: 3-(4,5-Dimethylthiazol-2-yl)-5-(3-carboxymethoxyphenyl)-2-(4-sulfophenyl)-2H-tetrazolium
Pam3Cys: Pam3Cys-Ser-(Lys)4
PGD2: Prostaglandin D2
PGE2: Prostaglandin E2
polyI:C: Polyinosinic–polycytidylic acid
pSTAT1: Signal transducer and activator of transcription 1
pSTAT3: Signal transducer and activator of transcription 3
RT-PCR: Reverse-transcribed polymerase chain reaction
SEM: Standard error of the mean
SOCS3: Suppressor of cytokine signaling 3
TMB: Tetramethylbenzidine
TNF: Tumor necrosis factor
